# Feasibility and Applicability of Implementing the Framework for Comprehensive Understanding of Structural Stigma in Mental Healthcare Systems: A Case Example of Nepal

**DOI:** 10.1111/hex.70170

**Published:** 2025-02-05

**Authors:** Dristy Gurung, Bhawana Subedi, Binita Acharya, Mani Neupane, Brandon A. Kohrt, Graham Thornicroft, Petra C. Gronholm

**Affiliations:** ^1^ Transcultural Psychosocial Organization Nepal (TPO Nepal) Kathmandu Nepal; ^2^ Health Services and Population Research Department Centre for Global Mental Health and Centre for Implementation Science, Institute of Psychiatry Psychology and Neuroscience (IoPPN), King's College London, De Crespigny Park London UK; ^3^ The George Washington University School of Medicine and Health Sciences, Center for Global Mental Health Equity Washington DC USA; ^4^ Department of Population Health London School of Hygiene and Tropical Medicine, Centre for Global Mental Health London UK

**Keywords:** healthcare systems, measurement framework, mental health, people with lived experience involvement, structural stigma

## Abstract

**Introduction:**

Mental health‐related structural stigma is a multifaceted issue that significantly impacts access to quality mental healthcare, particularly in low‐resource settings like Nepal. Therefore, there is a clear need to understand the complexities and identify gaps for targeted interventions through evaluations of various dimensions of structural stigma within healthcare systems. This study aimed to assess the feasibility and applicability of a mental health‐related structural stigma measurement framework through its implementation in Nepal's healthcare system.

**Methods:**

A mixed‐methods approach was employed, involving data mapping, key informant interviews and rating exercises with diverse stakeholders, including policymakers, health workers and people with lived experience (PWLEs). A visual analogue scale or Red/Amber/Green (RAG) rating scale was used to rate each indicator within the framework for the level of structural stigma based on the mapped information and their experiences. Data collection was carried out from May to June 2024.

**Results:**

Twenty key informants were interviewed for this exercise. Most indicators within the framework were endorsed as yellow, followed by red by participants referring to mid to high levels of structural stigma within the healthcare system. The findings also revealed that the stakeholders perceived the framework as acceptable and applicable for measuring mental health‐related structural stigma in the healthcare system. However, challenges were noted regarding the clarity of some indicators, limitations of the three‐coloured visual analogue rating and the need for comparator conditions.

**Conclusion:**

The study underscores the measurement framework's value as a tool for identifying and addressing structural stigma in a mental healthcare system in a low‐resource setting. Stakeholder engagement and contextual adaptation are crucial for its successful implementation. The insights gained can inform structural reforms and improve mental health service delivery, ultimately promoting greater equity and access for PWLEs.

**Patient Public Contribution:**

This framework being assessed in this study (FOCUS‐MHS) was developed through extensive consultation with People with Lived Experiences (PWLEs) in Nepal and globally with the Global Mental Health Peer Network along with other stakeholders. Identification of documents, policies and studies, along with qualitative information mapped within the indicators, was informed by PWLEs involved in local study sites and by policymakers and health administrators. The reflections of the study participants—PWLEs, health administrators and policymakers, have guided further refinement of the framework for future use.

## 1 Introduction

Stigma is understood as problems in knowledge, attitudes and behaviours that negatively affect people with lived experiences (PWLEs) of mental health conditions [1]. This definition portrays stigma as a socially constructed phenomenon enacted from an ‘in‐group’ towards an ‘out‐group’. However, these social constructs can be embedded within the structures of society that are reproduced to maintain and create further stigma and discrimination towards PWLEs [2, 3]. This form of stigma, also called ‘structural stigma’, reflects cultural norms, policies and practices that limit the opportunities of and access to care for PWLEs [2].

Stigma research has predominantly concentrated on the inter‐ and intra‐personal dimensions of stigma processes, with few studies examining structural stigma's nature, causes and consequences [2, 4]. This could be due to structural stigma being a multifaceted issue that involves various societal and institutional layers, making it challenging to capture comprehensively with a single instrument [5]. This has resulted in fewer tools explicitly designed to assess structural stigma.

The varied conceptualisations and diverse measures of structural stigma have posed significant challenges for uniformity in measuring and comparing structural stigma in different settings [6]. Furthermore, these inconsistencies prevent the development of targeted interventions to reduce structural stigma, as the complexities underlying the structural stigma processes remain inadequately understood. This is specifically true in a complex system such as the healthcare system setting, which consists of multiple interconnected components and actors [7]. While the core components of healthcare systems are broadly consistent across contexts, their culture, practices and norms vary. Comprehending the dynamics underlying structural stigma is difficult without a nuanced understanding of the systemic cultures and practices.

**Figure 1:**
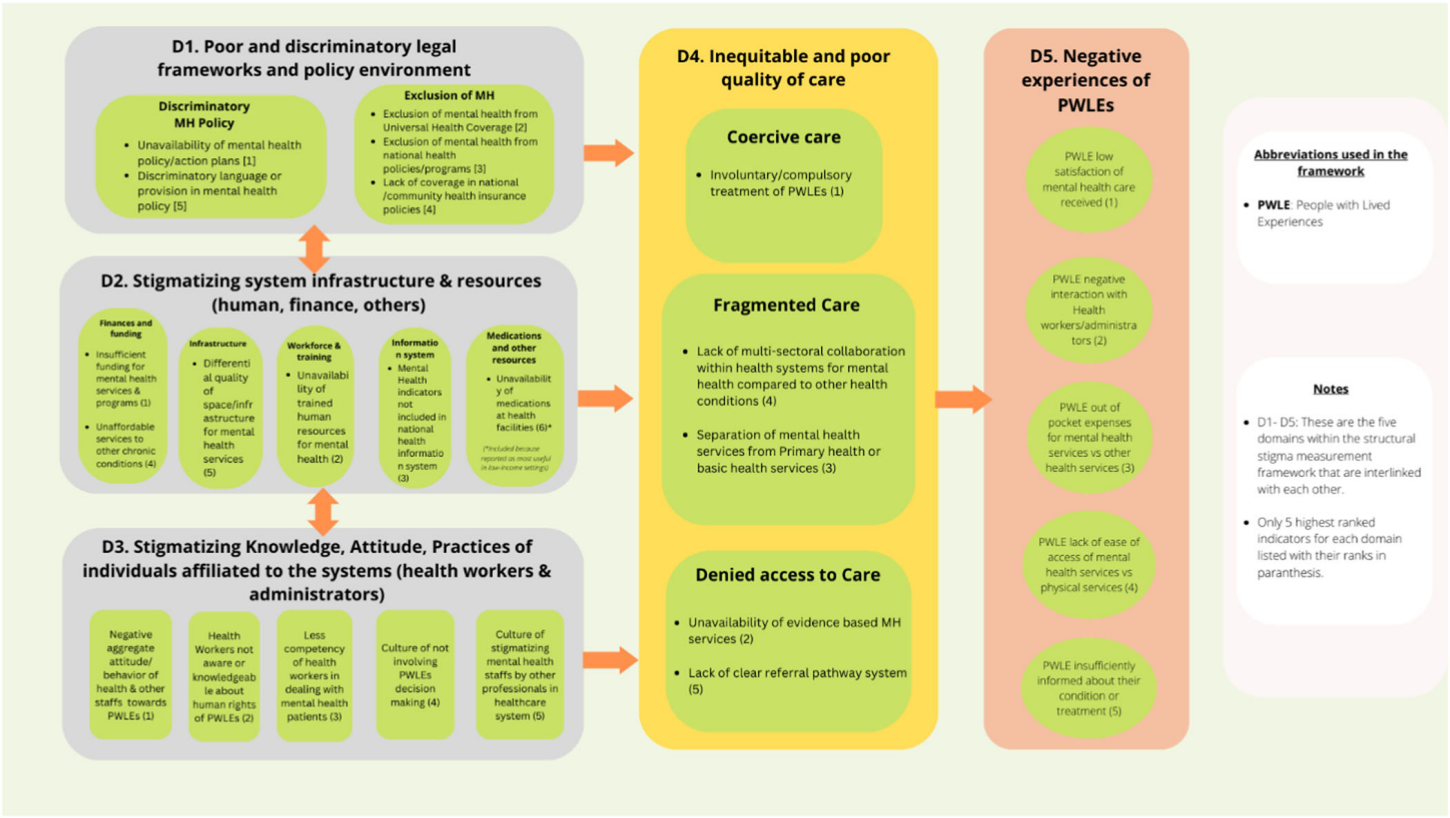
Domains and indicators in FOCUS‐MHS framework.

To introduce an innovative approach to examining structural stigma within healthcare systems, our research group developed a structural stigma measurement framework called the Framework for Comprehensive Understanding of Structural Stigma in Mental Health Systems (FOCUS‐MHS) using a 3‐round modified Delphi approach involving mental health stigma experts (researchers, PWLEs, and policymakers) [6]. The FOCUS‐MHS framework consists of five interconnected key domains, illustrated in Figure 1: D1: poor and discriminatory legal framework and policy environment, D2: stigmatising system infrastructure and resource allocations, D3: aggregate stigmatising attitude and practices of healthcare system personnel, D4: inequitable and poor quality of care and D5: negative experiences of PWLEs. Each domain within the framework includes five to six indicators.

In the current study, we aimed to assess the feasibility and applicability of implementing FOCUS‐MHS using Nepal as an example setting. Feasibility refers to the ease of data collection without imposing undue burdens with a focus on data availability and trustworthiness and the ease of understanding rating systems. Applicability refers to the contextual fit to the healthcare system setting and the relevancy of the information to different stakeholders. Nepal was selected because it represents a low‐resource healthcare system setting that has recently started scaling up the community mental healthcare programme and has launched a mental health strategy and action plan. However, mental health‐related stigma has been identified as a barrier to the scale‐up [8, 9]. Our results are intended to inform the further development of a flexible strategy for measuring structural stigma in various healthcare system settings.

## 2 Methods

### 2.1 Setting

Nepal exemplifies many low‐resource settings that are struggling to expand mental health services due to policy, legislative and logistical barriers [10]. Nepal's current health system structure is reflected through the federal government systems, which consist of the federal, provincial and local levels of administration. At the federal level, the Ministry of Health and Population (MoHP) oversees overall health policy and planning and is responsible for the governance, planning and implementation of mental health services through various departments and units [11]. For a country of 31 million people, Nepal's only national mental hospital with 50‐bed capacity is in Kathmandu, the capital city. Although healthcare systems have multiple tiers of structure (at federal, provincial, district and municipality levels), policy development, planning and resource allocations for mental health are carried out in top‐down approaches.

### 2.2 Data Mapping Exercise

The first step in implementing FOCUS‐MHS was to map relevant data readily available through online sources for each indicator under its five domains. Two researchers (D.G. and B.S.) charted information from reviews of mental health stigma literature [12] and mental health‐related health and social policies [11] into the indicators using an Excel sheet. Next, we searched Google Scholar for further published peer‐reviewed or grey literature for missing information or information requiring updates. Relevant government websites and their reports were also scoped to identify information for each indicator. The data mapping exercise was conducted from March 2024 to April 2024.

### 2.3 Key Informant Interviews (KIIs)

#### 2.3.1 Sampling

A total of 20 experts (5 females and 15 males) participated in the study, selected through purposive sampling based on the following expertise:

i. PWLEs and service user advocates (*n* = 5; 1 female)
ii. Policymakers and health system leaders (*n* = 6; 1 female)
iii. Mental health service providers/health workers (*n* = 2; 1 female)
iv. Mental health development partners (I/NGOs) (*n* = 7; 2 females)

In addition to the 20 participants, 2 experts did not respond and 5 were unavailable during the data collection timeframe (7 May to 16 June 2024). The study's sample size was determined by the data adequacy for the aim of the study (from debriefing notes of researchers), the selection of expert participants most relevant to the study and the in‐depth/rich information from each participant.

#### 2.3.2 Data Collection

Participants were first approached through emails or telephone, and the study information sheet and consent form were sent for review and signing before interviews. Most interviews were conducted via Zoom, and some PWLE interviews were conducted in person. Interviews were audio‐recorded, conducted in Nepali, and lasted 1.5–2 h. Data adequacy was assessed through reflexivity and debriefing notes post‐interviews [13]. The semi‐structured interview guide was developed using a priori themes covering a range of feasibility and applicability topics (see Table 1).

**Table 1:** Topics covered during KIIs.

i. Understanding and examples of mental health‐related structural stigma in healthcare systems ii. Review and initial impression of the FOCUS‐MHS framework, domains and indicators iii. Recency, relevancy, accuracy and any gaps in the information mapped in each indicator, and how the information gap can be filled iv. Rating the indicators based on available information, experts' experiences and their justification for the rating, along with examples of situations that could change the ratings v. Discussion of the feasibility of using the rating system and recommendations for improving the assessment process vi. Reflections/comments/thoughts on the applicability and usefulness of the FOCUS‐MHS framework in Nepal's healthcare systems

The Red‐Amber‐Green (RAG) rating system was used as a visual analogue scale to measure each indicator's structural stigma level. This system is widely used in health systems to appraise risks to patients [14] and to visually summarise the quality and performance of indicators [15]. Key advantages of the RAG rating system include its versatile application across various indicators, enhanced accountability over time and ability to rapidly identify areas needing attention (red), improvement (amber) or which are performing well (green) [16, 17]. One key consideration when applying indicators is the type of stakeholders involved [18]. As applied to FOCUS‐MHS, the RAG rating system simplifies communication with policymakers, health leaders and PWLEs through visual clarity.

The criteria used for the rating were based on information mapped and my knowledge/experience: Red (ongoing structural stigma in mental health with no efforts to address it); Amber (some structural stigma in mental health, but some efforts to address it); and Green (no structural stigma in mental health, with mechanisms in place to maintain this).

### 2.4 Data Management and Analysis

The percent endorsement of Red/Amber/Green for each indicator was analysed descriptively using MS Excel. The qualitative data analysis used the framework approach [19], where a priori themes based on topics explored and domains/indicators within the FOCUS‐MHS framework were generated in an Excel sheet. Researchers (D.G., B.S. and B.A.) fluent in both Nepali and English transcribed and translated the recorded interviews. Three researchers deductively coded, charted and indexed interview transcripts in the Excel sheet that included the a priori themes in the rows (e.g., their understanding/experiences of mental health‐related structural stigma, data gaps in terms of recency/relevancy/accuracy, the feasibility of getting information for the indicator, etc. for each indicator), and respondent ID in the columns. The team logged memos and discussed any issues, possible solutions and critical reflections during the processes.

## 3 Results

**Figure 2:**
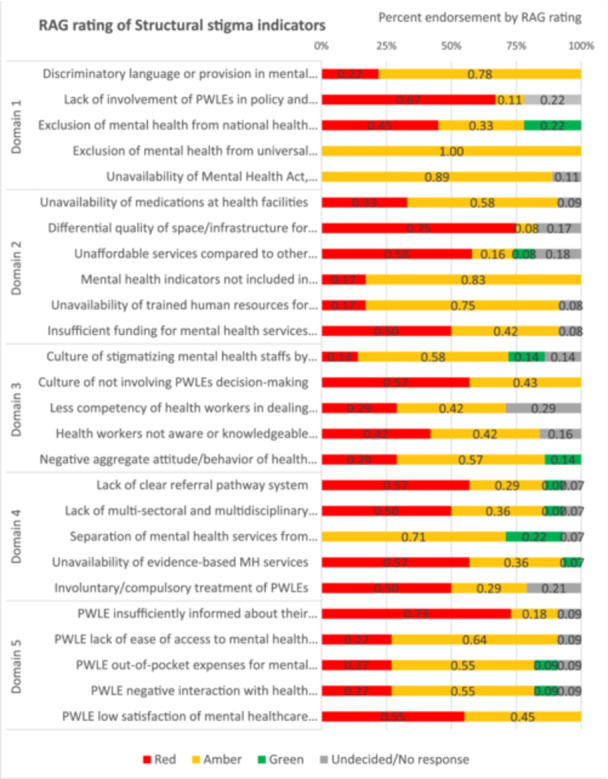
Endorsement of structural stigma indicators by stakeholders in Nepal.

Table 2 summarises the information available for each indicator, sources of information from the data mapping or as recommended by the participants and relevant quotes from the KIIs on the indicator's status in Nepal's healthcare system. Similarly, the level of structural stigma, as indicated by the percentage endorsement using RAG rating, including the non‐response and indecisiveness, is visually illustrated in Figure 2.

**Table 2:** Summary of mapped information, its sources and relevant quotes from KIIs.

Domains/indicators	Information from the mapping exercise and KIIs used for rating	Information sources identified or suggested	Relevant quotes from participants on indicators and their rating
Domain 1	Poor and discriminatory legal framework and policy environment		
Unavailability of Mental Health Act, policy/action plan	‐ The government of Nepal drafted a national mental health policy in 2017, but the cabinet minister did not approve it, and hence, it never came into existence. ‐ National Mental Health Strategy and Action Plan 2077 was formulated to provide easy, accessible, and quality mental health services to the people. ‐ No mental health act formulated in Nepal to date. ‐ Gandaki and Karnali province has included mental health in its own provincial health policy.	‐ Policy review ‐ WHO Mental Health Atlas indicators ‐ Survey/interviews with policy stakeholders	A participant highlighted what should be present for the indicator ‘exclusion of mental health from universal health coverage’ to turn green: ‘Service availability should be maintained. Right now, only 23% of the population receives mental services‐ even if we go by the best estimate. In all of primary healthcare, there needs to be at least two trained human resources available in the primary healthcare facilities at all times, medicines should not be disrupted for more than seven days in a year, and district hospitals should be the first level of referral with at least one psychiatrist to be available as a trainer/supervisor and also provide tele‐mental health’. *(Male, development partner/mental health specialist)* A participant on why they were undecided between red and yellow for the indicator ‘unavailability of mental health act, policies/action plan’: ‘The provincial and local governments should develop mental health strategies based on their provincial scenario. All levels of government should prioritize mental health and implement the strategies’ *(Female, development partner).*
Exclusion of mental health from universal health coverage	‐ Mental health included in the Basic Healthcare Service, 2075, to ensure that health services are easily accessible and available free of cost. ‐ The Social Health Security Scheme includes the medical management of conversion disorder, severe depression, bipolar disorder, epilepsy and schizophrenia. However, psychosocial management/therapies are not listed in the scheme. ‐ The national health insurance programme was initiated in 2017 based on the Health Insurance Act and ensured its implementation in all 77 districts by the year 2022. However, this is yet to be achieved.	‐ Policy review ‐ In‐depth review of insurance policy documents	
Exclusion of mental health from national health policies/programmes	‐ The Public Health Service Act of 2018 includes the services related to mental health as free basic health services. ‐ Mental health agenda is included sparsely in national health policies and acts. ‐ National Health Policy 2019 addresses mental health as a concern and ensures access to mental health and psychosocial services from local health facilities by promoting knowledge and skills transfer in primary hospitals. ‐ Mental health is rarely included in wider national public health programmes such as maternal/child health and HIV/NCD programmes.	‐ Policy review ‐ Survey/interviews with stakeholders	
Lack of involvement of PWLEs in policy and programme development, implementations and evaluations	A qualitative study conducted among service users and caregivers in Nepal stated that PWLEs and advocates are rarely involved in policy and programme development/implementation/evaluation phases. Any involvement is only tokenistic, with feedback rarely sought or considered.	‐ Qualitative interviews with PWLEs/advocates ‐ Observations of stakeholders narrated during KIIs	
Discriminatory language or provision in mental health policy	A scoping review of health and social policies of Nepal showed some progress had been made in revising stigmatising language, labelling terms such as 'baulaaeko' (insane), imprecise language (mental disease, drug abuse), devaluing terms (mentally incapable/handicapped, mentally incapacitated) and dated terminology (mental asylum) persists in several policies. ‐ It also noted discriminatory provisions such as lack of legal capacity to consent. ‐ Significant discrepancies between provisions between and within policies were noted.	‐ Policy review	
Domain 2	Stigmatising system infrastructure and resource allocations		
Insufficient funding for mental health services and programmes	A study from 2020 reflected only 0.2% of the budget allocated to mental health out of 6.15 for the overall health budget ‐ The government has allocated less than 1% of total national health expenditure, with a significant proportion directed towards mental hospitals. As per the WHO Report 2022, the estimated annual budget for mental health intervention (excluding human resources and hospital operations) is USD 1.5 million, that is, NRS 199,955,421 (approx 20 Cr.). The per capita public funds allocated for mental health is approximately 0.05 USD that is, NRs 6.67.	‐ National health accounts ‐ WHO report ‐ EDCD ‐ Line Ministry Budget Information System (LMBIS)	A participant from a development partner agency highlighted how the differential quality exists explicitly at the hospitals that provide both physical and mental health services: ‘Where it does differ is in the hospitals where there are in/outpatient mental health services. Usually, the OPD (Outpatient Department) for mental health is allocated in a far‐off corner where no one wants to go. The psychiatric wards are typically underground or far from the main facilities, with limited lights and mouldy rooms. Suitable buildings with good infrastructures are not reserved for mental health wards; they are reserved for other conditions. In addition, if new beds are bought, they are handed over to other wards, and the older beds are allocated to psychiatric wards. We have observed infrastructural discrimination in such ways’ *(male, development partner/mental health specialist).* A government official discussed the competing needs within the healthcare system, resulting in a limited mental health budget: ‘We are still struggling with infectious diseases such as malaria and leprosy elimination. Our targets and activities are focused on this. If you look at the Ministry of Health‐ it has a Department of Health Services, and the department has ten divisions, one of which is the NCD and mental health unit. The NCD and mental health are put together even in that one division. So, you can imagine how much of our budget is for mental health’. *(Female, policy maker/health system leader)* A participant on cut down of mental health programme budget: ‘When the government goes into crisis, they target to cut down the mental health budget rather than from budget allocated to physical health conditions. I don't think the gov understands the importance of mental health’. *(Male, development partner)* A participant at the government level outlining issues in the procurement process of psychotropic drugs: *For basic healthcare, we have classified the medicines out of the 98 essential healthcare medicines, ones that the federal government can purchase, ones that the province level can purchase, and ones that the local level can purchase. So, what happened was that the purchase of mental health medications is listed under the province's government. The challenge is that the provincial government says that they don't receive enough budget. However, they don't have data on quantification‐ how many cases are there and how many medications are required‐ they don't have this information. It would be easy if the local government could inform the province that they need these medications of these dosages. Because the medications are not that expensive, the federal government tells them that the budget they have sent them is enough. Another issue is that the local level cannot purchase psychotropic medications (I don't know if it is due to legal issues surrounding psychotropic drugs). Because they cannot purchase it themselves, the problem is that they must depend on the quantities sent by the province. But then the province doesn't know what quantities it is required for. A lot of local government bodies are lobbying for them to be able to purchase mental health medications. However, the basic health care package is like a law, so we haven't been able to amend it quickly. That's our gap. (Female, policy maker/health system leader)*
Unavailability of trained human resources for mental health	The trained healthcare providers as of the year 2022 are psychiatrists (200), psychologists (30), psychiatric nurses (75), psychosocial counsellors (1300), prescribers trained at local health facilities (more than 1700), female community health volunteers (938). The accurate number of trained healthcare providers for mental health is not made accessible to the general public through the official government website; hence, the data mentioned from different studies or WHO may be inaccurate as compared to the current scenario.	‐ WHO report ‐ National Health Training Center (NHTC) ‐ MoHP ‐ Psychiatric association data log ‐ Nursing association data log	
Mental health indicators not included in national health information system	The Health Management Information System (HMIS) reports and records all the delivered health services from all levels of health system of Nepal. In the case of mental health, the indicators related to mental health in HMIS are included with archaic indicators in HMIS 9.3. Recently, new NCD and mental health indicators have been developed that logs information on diagnosis and treatment of priority mental health conditions and disaggregates gender and age. However, training and implementation of these new indicators are pending.	‐ HMIS 2070 ‐ Revised HMIS indicator 2078	
Unaffordable services compared to other chronic conditions	The findings from the NHRC National Survey 2020 found the average expenditure on treatment of mental disorders in the past 12 months was found to be NRs 16,053; expense on transport and other costs associated with seeking treatment was NRs 4146 and NRs 3460 respectively. A study conducted on out‐of‐pocket expenditure associated with depression found a total mean expenditure of $118 USD (NRs 15,730) per year out‐of‐pocket on healthcare. This amount represents a substantial proportion of household budgets, especially considering that the median annual income for adults with depression in the area is estimated to be $501 USD (NRs 66,785).	‐ National Mental Health Survey ‐ Costing and Cost‐effectiveness studies	
Differential quality of space/infrastructure for mental health services	Studies have found the lack of separate/private space for counselling as a barrier to mental health services. The ongoing study on mental health (RESHAPE) [20] in the three districts of Gandaki Province has the data of 21/30 Health facilities in 8 municipalities did not have the OPD or counselling space required to deliver mental health services.	‐ Findings from WHO Quality Rights toolkit ‐ Qualitative studies and site observations ‐ Health facilities observation	
Unavailability of medications at health facilities	The data from the RESHAPE study found that essential medicines like antidepressants/SSRIs, antipsychotic medications, and benzodiazepines were continuously unavailable for more than 4 months in 3, 9 and 16 health facilities out of 24, respectively. Additionally, 24 HFs rated ease of procurement of psychotropic medications on average 3.7 compared to procurement of other drugs (average 6.9) on a scale of 1–10 where 10 was very easy to procure while 1 was very difficult to procure.	‐ Government Logistic management data system ‐ Observation ‐ Health facility store logs	
Domain 3	Aggregate stigmatising attitudes and practices of healthcare system personnel		
Negative aggregate attitude/behaviour of health and other staff towards PWLEs	Studies conducted on stigma and barriers to mental health services in Nepal have identified stigma among health workers as a significant barrier to service delivery. Health workers often perceive a risk of personal harm and violence and fear of losing social prestige in the community when treating people with lived experiences (PWLE). However, there is a lack of data addressing the stigma of healthcare staff within hospitals/healthcare settings.	‐ Use of stigma attitude tools during training ‐ In‐depth interviews with PWLEs	A health coordinator from a rural municipality reflects on the indicator ‘culture of not involving PWLEs in the decision‐making process’: ‘The patient who recovered from the physical health problem is trusted fully as compared to those who have mental health conditions. There is still a huge stigma towards people who are recovering from mental health conditions, that they aren't capable enough to make the decision’. *(Male, Health coordinator from rural municipality)* A health worker on rating the indicator ‘negative attitude of health systems staff’ as red based on their experience with other health workers in their facility: ‘When the Alcohol Use Disorder (AUD) patient comes to our health facilities, my colleague stays away from them or tells the patient to stay outside. If the psychosis patient visits health facilities, they don't want to handle that case. The Health Workers used to give less time to that patient, but it may have been due to a fear of harm to them’. *(Male, Health facility Incharge/health worker)* Reflection by a participant on the indicator ‘Health system professionals not aware about human rights of PWLEs’: ‘There is a huge gap as these are not researched, but based on my observations, I would give a red rating to this. There aren't many initiatives to address this issue, and we need to do quite a lot about this. Even as part of the mental health training, where have we talked about human rights? Even in module 1 and module 2 training of health workers, human rights issues are not addressed. We train them on basic communication skills, therapeutic skills, and medications, but we haven't incorporated human rights topics in these trainings for health workers’. *(Male, Development partner)*
Health workers not aware or knowledgeable about the human rights of PWLEs	There is not any reported data on health workers' knowledge or awareness level about the human rights of PWLEs. Limited training for health workers or administrators on the rights of PWLEs. A few quality rights trainings organised by I/NGOs.	‐ No identified sources for data	
Less competency of health workers in dealing with PWLEs	The competency measure of health workers in dealing with PWLEs was shown by a study conducted in Nepal along with two other countries that found health workers' incompetency in diagnosis, addressing confidentiality, involving family members in care, and assessing suicide risk. Similarly, the Enhancing Assessment of Common Therapeutic Factors (ENACT) tool that assesses the diagnostic accuracy of mental illness showed the result of only 10 of 29 accurately diagnosed by the PCPs. However, there was not any data available on the competency measures of other health staff (nurses, assistants, administrators) in the healthcare setting.	‐ Use of ENACT or other competency‐based tools ‐ PWLE observations and interviews	
Culture of not involving PWLEs decision‐making	A qualitative study with PWLEs from Nepal found that they were not involved in major decision‐making processes such as in discussions about medication and treatment options	‐ Qualitative interviews with PWLEs	
Culture of stigmatising mental health staff by other professionals in healthcare systems	Qualitative studies have shown health professionals often face the stigma from their colleagues whenever they used to work in the psychiatric ward. They were given names as 'psycho doctors' just because they treated the MH patients.	‐ Qualitative interviews with health professionals	
Domain 4	Inequitable and poor quality of care		
Involuntary/compulsory treatment of PWLEs	The PWLEs shared they were prescribed unnecessary medicine for their conditions because the doctor would often get profit from the pharmaceutical company for prescribing those medicines. Some PWLEs mentioned they were subjected to involuntary confinements and hospitalisations for their conditions even though they asked to be released.	‐ Focus group discussion among PWLEs ‐ In‐depth interviews with PWLEs	A health coordinator on ‘involuntary/compulsory treatment’ yellow mentioned: ‘I think it depends on the case. The health workers want the patient to get better, and it depends on how the patient is. If the patient is violent, causing harm to others and destroying property, then they should even be locked up. (Interviewer: So, the patient can be treated involuntarily and even imprisoned for their safety?) Yes, that's why doctors make those decisions’. *(Male, Municipality health coordinator)* A mental health specialist rating the same indicator ‘involuntary/compulsory treatment’ red: ‘In most cases, family members of PWLEs are not involved by clinicians in decision‐making‐ there isn't a practice for that, even from qualified practitioners. Prescribing a lot of unnecessary drugs and over‐sedation‐ are the issues. This is even true for who we call mental health specialists. This is a big area that we can work on. If you sell many drugs, you make a lot of profits, which is true in many profit‐making institutions. This is one aspect. The other aspect is that if the patient is sedated‐ it's much easier for ward staff on duty. These are some of the malpractices I have observed as an insider’. *(Male, mental health specialist)* A participant on why they rated the indicator ‘lack of clear referral pathway’ as red: ‘In the case of maternal health, Skilled Birth Attendant has a separate slip with them if they cannot manage the case. The referred hospital will know where the case came from and what the patient's situation is. The HMIS also has reporting of referred cases for maternal health. But in the case of mental health, there aren't any referral slips available, and there isn't any system to track whether the referred patient has received treatment. There are many losses to follow‐up cases because of the weak referral mechanism’. *(Female, development partner)*
Unavailability of evidence‐based MH services	The integration of mental health into basic healthcare programmes was based on evidence from the PRIME project that adapted the WHO's mhGAP intervention to Nepal's context. WHO looking at evidence of tele‐psychiatry to inform government. No other information on whether mental health and psychosocial programmes are evidence‐based. Mental health research and data quality from HMIS is limited and so less likely to have evidence‐based mental health programmes.	‐ Government policy introductions ‐ NGO reports	
Separation of mental health services from Primary health or basic health services	Public Health Act, 2075, has included mental health in basic healthcare services. Looking at the current scenario, the integration of mental health is in the scale‐up process. As per the WHO Report 2022, the GoN‐ MoHP has now integrated the mental health service in 35 of the country's 77 districts through PHC and district hospitals. Primary health care (PHC) providers were taught to diagnose, identify, and manage basic mental health issues. At least one medical doctor in Nepal's 35 district hospitals was trained in mental health.	‐ WHO report ‐ Policy review	
Lack of multi‐sectoral and multidisciplinary team collaboration within health systems for mental health compared to other services	There is not any reported information available on how the different levels of government healthcare systems collaborate within different departments or institutions for mental health programmes	‐ Observations and qualitative interviews with stakeholders	
Lack of clear referral pathway system	A study from Nepal [21] highlights the lack of clear referral mechanisms from primary to tertiary care, which is one of the barriers to the identification and treatment of mental health patients. Besides that, there is not any information available about the two‐way referral pathways.	‐ Research study	
Domain 5	Negative experiences of PWLEs		
PWLE low satisfaction of mental healthcare received	The PWLEs mentioned they had low satisfaction of care received from health facilities and hospitals due to the lack of medicines, health workers' rude behaviours towards them and lack of privacy (no separate space to share their problems openly)	‐ FGD with the PWLE ‐ PWLE service satisfaction surveys	PWLEs reflecting on the difficulties in accessing mental health care: ‘If the doctor gives more time and asks the patient questions, they could identify mental health problems. If not, the patient must go to many places for their diagnosis, ultimately increasing expenses. People visit Dhami chakra (traditional healers) and spend money; they go to the district hospital, but the GP cannot identify the mental health problems. In this process, they spend a lot of money’. *(Female, PWLE)* A PWLE shared his negative experience of hospitalisation: ‘In the hospital where I was admitted, I remember this lady who told us we were prisoners there. They never told us why we were there, for how long, why we were imprisoned there without our choice. I used to think about it and felt very bad at that time. I have talked to many people who have faced similar experiences’. *(Male, PWLE)* A PWLE on ease of access to services: ‘There was a person in our community who ran away from home‐ he was naked, had no slippers, was vulnerable to himself, and was aggressive to others. We took him to the government hospital, but they said that there's no bed for people having a mental health problem and asked us to leave after a day of admission. They would have treated him if he had been taken to a private hospital, but they couldn't afford it. At the government hospital, we somehow managed to keep him there for a night, but they kept him tied up the whole night, and the next day, the hospital didn't want to keep him there. So, we had to send him to Kathmandu the next day by making him unconscious. What can we say now? (laugh). The access to services is zero’. *(Male, PWLE)* A PWLE on lack of communication and sufficient information by service providers: ‘They have 4/5 doctors in the same room and just write the medicines without listening to patients. They ask them to come again after one week or 15 days. That's all based on their (doctor's) assumptions, not what patients told them. It's like they can do whatever they want’ *(Male, PWLE)*
PWLE negative interaction with health workers/administrators	Some PWLEs shared that the doctors and other staff tease them while visiting for check‐ups. A few of the AUD patients shared the doctor made inappropriate jokes like how many bottles of alcohol they consumed during consultation. Furthermore, they added that the doctors would not listen to them properly while sharing their problems.	‐ Qualitative interviews with PWLEs ‐ PWLE quality of service surveys	
PWLE out‐of‐pocket expenses for mental health services	PWLEs have shared they have to travel and buy medicine from the city with their own money because of the medicine's unavailability at the health facility	‐ FGD and qualitative interviews with PWLEs ‐ Costing and cost‐effectiveness studies	
PWLE lack of ease of access to mental health services	PWLEs shared they were unable to find medicine when prescribed by the doctor and had to go to multiple places in search of medicine for mental health problems. They shared that medicines are not as easily available for mental health conditions as compared to physical health conditions.	‐ FGD and qualitative interviews with PWLEs	
PWLE insufficiently informed about their condition or treatment	PWLEs mentioned that health workers do not give enough time to listen to their problems or inform them properly about their problems	‐ Qualitative interviews and FGDs with PWLEs	

### 3.1 Domain 1: Discriminatory Legal Framework and Policy Environment

The indicator ‘exclusion of mental health from universal health coverage’ had the most consistent rating (100% rated as amber). All participants indicated that the inclusion of mental healthcare services within the basic healthcare package and the government's health insurance scheme clearly showed attempts made at inclusion. However, they mentioned substantial work was still necessary for the indicator to turn green due to the lack of scheme coverage, geographically limited implementation of mental health programmes within primary healthcare services and the lack of comprehensive mental health services (e.g., psychosocial counselling, evidence‐based therapies) within basic health and insurance packages.

Indicators such as ‘unavailability of mental health act and policies’ and ‘discriminatory language and provisions in mental health policies’ also had a higher percentage of amber rating, indicating some levels of discrimination but also attempts made to address them. For example, almost all participants mentioned the drafting of national mental health strategies and action plans as a huge step forward. Still, without a mental health law that impacts PWLE's services and rights, the indicator has yet to turn green.

### 3.2 Domain 2: Stigmatising System Infrastructure and Resources

Most participants rated ‘unavailability of trained human resources for mental health’ (75%) and ‘mental health indicators not included in national health information systems’ (83%) as amber, as in these areas, the government had realised potential gaps and initiated mechanisms such as prioritising the mental health training for primary healthcare workers, initiating health administrators training for the implementation of mental health programmes and incorporating additional indicators for better understanding national‐level mental health data. Nevertheless, they raised concerns that much was needed to turn the indicator green, such as increasing the number of trained health workers at health facilities and creating specialist positions at district hospitals, ensuring not just clinical team availability but also the therapists/counsellor and nurses' availability to provide holistic psychosocial support and implementing the newly adapted mental health indicators in all tiers of healthcare.

The ‘differential quality of space/infrastructure’ indicator had a higher rating of red (75%). In comparison, some participants (17%) rated it between red and amber, given the unavailability of specified beds or in‐patient units within the larger provincial and district hospitals. Similarly, participants highlighted that although there were infrastructural limitations at the primary healthcare facilities, some municipalities and health facilities with motivated health professionals and administrations had created ad hoc confidential spaces within their limited infrastructure for the provision of mental health services (e.g., meeting rooms within ward administrative offices or medication dispensary room), highlighting the lack of space as a stigma issue.

Participants seemed ambivalent towards indicators such as ‘insufficient funding for mental health’ and ‘unavailability of medications’, with ratings split between red and amber. In terms of funding, all participants agreed that the budget was insufficient to implement mental health policies and programmes. Some participants emphasised that mental health program funding mostly came from development partners, leading to drastic changes (increases or decreases) in mental health expenses from 1 year to another, as such funding is short‐term and unsustainable.

Ratings for ‘unavailability of medications at health facilities’ were distributed between red and amber, mainly due to limited clarity on whether it was a supply chain problem (if so, are there supply issues also for medications for other health conditions) or is restricted supply chain management caused by issues with mental health‐specific policies. While most agreed that including psychotropic medications in the free drug list was a huge step forward, its implementation was lacking as the medicines could only be supplied to health facilities with trained health workers. Even then, the supply of medicines was ad hoc, without proper evaluation of prevalence, catchment population or need (due to problems in recording mental health indicators), resulting in either an over‐ or undersupply of medications. A highlighted structural stigma issue was a policy on authorising the purchase of psychotropic medications. While most drugs on the free drug list could be authorised for purchase by local government (municipalities), purchasing psychotropic drugs was only permitted by the provincial and federal government bodies, leading to lengthy procurement and supply time.

Participants also seemed unsure about ‘unaffordable services compared to other chronic conditions’, with ratings distributed between red (58%), amber (16%), green (8%) and undecided (18%). Participants found the indicator difficult to rate due to a lack of aggregated representative data on out‐of‐pocket (OOP) costs for mental health compared with other physical health conditions.

### 3.3 Domain 3: Stigmatising Knowledge, Attitude and Practices of Individuals Affiliated With the Health Systems (Health Workers and Administrators)

These indicators had the most variable ratings compared to other domains, although most ratings were between red and amber. The ‘culture of not involving PWLEs in decision‐making’ had a higher red rating (57% vs. 43% amber). In comparison, ‘negative aggregate attitude/behaviour of health systems staff’ (57% amber, 29% red, 14% green), ‘culture of stigmatising mental health staff’ (58% amber, 14% red, green and undecided each) and ‘less competency of health workers’ (42% amber, 29% for both red and undecided) had a higher proportion of amber rating compared to red and green. As most indicators in this domain had no aggregate data or information that could be mapped, the ratings were based on personal experiences, which varied between participants.

The ‘health systems staff is not aware/knowledgeable about human rights of PWLEs’ indicator had comparable ratings for red and amber (42%), while 16% were undecided between red and amber. The varying opinions reflect the participants' experiences. For example, a health coordinator thought the indicator was irrelevant because health professionals were not lawyers and did not need to know the human and legal rights of PWLEs. Some participants, however, believed that the indicator was very relevant and necessary and rated it amber as some development partners had conducted WHO Quality Rights training [22]. Some participants rated it red, as the lack of data or information for the mental health indicator was considered to reflect structural stigma, and a lot was needed to turn the indicator green.

### 3.4 Domain 4: Inequitable and Poor Quality of Care

Like the indicators in Domain 3, these indicators had limited data from published literature, so the information was mainly derived from qualitative studies conducted in specific geographic or healthcare settings. As such, indicators like ‘involuntary/compulsory treatments’, ‘separation of mental health from primary healthcare’ and ‘lack of collaborations’ had higher non‐response rates compared to other indicators. Nevertheless, all indicators within this domain except ‘separation of mental health from primary healthcare’ were rated mainly as red, implying higher manifestations of structural stigma in this domain.

For example, 50% of participants rated ‘involuntary/compulsory treatment of PWLEs’ as red. This was corroborated by a mental health specialist rating the indicator red due to unnecessary sedation and overprescription, even by specialist practitioners. In contrast, 29% rated it amber by those who perceived improvement in restraining or compulsory admittance practices over time, occurring only when justified by the nature of the condition. About 21% had no response due to lack of information

Most participants also rated the ‘unavailability of evidence‐based mental health services’ red, given the government not prioritising evidence generation (evidenced by no mental health data for use in resource allocation), using a political agenda rather than evidence to inform policies, and the information lacking for most indicators within the framework. Some participants, however, highlighted the adaptation and implementation of WHO's mhGAP program [23] as a noteworthy government step and therefore rated it yellow.

The ‘lack of multi‐sectoral and inter‐disciplinary team collaboration for mental health services’ had more varied ratings, with 50% red, 36% amber, 7% green and 7% undecided/no response. Those with amber or green ratings highlighted government attempts to bring together different stakeholders for policy and planning. They also highlighted that such collaborations were more common in mental health than other health conditions, making it a comparatively lesser structural issue for mental health. Nevertheless, some participants considered collaboration and communication between different tiers of government—federal, provincial and local—challenging for mental health and therefore rated this red. Medication supply problems were given as an example of lacking collaboration between different tiers of government bodies and institutions, such as the health directorate office, health training centre and health logistics management centre.

The ‘lack of clear referral pathway’ indicator, too, had mostly red ratings (57%). Participants highlighted lacking referral mechanisms between primary healthcare facilities and general or specialist hospitals, compared to safe motherhood programmes.

### 3.5 Domain 5: Negative Experiences of PWLEs

Most information mapped to these indicators came from either FGDs, expert consultations with PWLEs during the framework's development or qualitative studies on barriers to care in Nepal. Most participants (55%) rated ‘low satisfaction of mental health care received’ as red, while the rest rated it as amber. PWLE participants especially resonated with the information mapped on the indicator and shared their own experiences of dissatisfaction with the care received. This ranged from the unavailability of medications resulting in having to purchase medicines from private pharmacies, health workers not listening and communicating like a ‘robot’, lack of freedom during hospitalisations and being treated as prisoners, and the troublesome/annoying process of using services like insurance schemes.

Most participants (55%) rated ‘negative interaction with health system staff’ as amber, indicating some progress in this area. However, several initiatives were needed to turn the indicator green, given issues like the use of stigmatising words towards patients, teasing patients, making snide comments about the patient while talking amongst themselves, commenting on them looking normal when trying to access disability services, being ignored or not taken seriously and being judged. The government initiatives to integrate mental health into essential healthcare services, with primary healthcare workers trained to provide free basic mental health treatment, was the reason behind most participants' amber ratings for ‘high OOP expenses for services’ (55%) and ‘lack of ease of access to mental health services’ (64%). However, some PWLEs rated these indicators ‘amber’ due to experiencing difficulties in accessing care, such as accessing services from multiple providers and spending lots of money before receiving a diagnosis or treatment.

The final indicator, ‘PWLEs insufficiently informed about their conditions and treatment’, was rated red by most (73%). PWLEs shared instances where they observed no two‐way communication between service providers and patients in a government hospital's psychiatric outpatient ward, where service providers prescribed medications and asked patients to attend follow‐up without considering the patient's or their caregiver's opinions.

## 4 Discussion

This study explored the feasibility and applicability of implementing FOCUS‐MHS, a mental health structural stigma measurement framework, in Nepal's healthcare system context. The results also report the RAG rating of each indicator within the five FOCUS‐MHS domains by the expert stakeholders based on the information mapped and their knowledge and experiences.

Applying the framework in Nepal's healthcare system revealed important areas where structural stigma was pervasive. Although Nepal's health system has seen increased attention towards mental health through the integration of mental health into the basic healthcare package [24] and through the drafting of a mental health strategy and action plan [25], the findings show numerous structural gaps, as most of the indicators were rated amber, followed by red. However, the findings need to be interpreted cautiously as understanding the level of structural stigma in Nepal's health system was not the study's primary aim, so the percentages are reflected from the small sample size (*n* = 20).

In Domain 1, although there exists a mental health action plan and strategies, in the absence of a mental health act, participants mentioned that all plans would remain in documents without proper implementation—reflecting the current scenario. This is especially true in integrating mental health into national health policies, which has rarely been translated into programmes and activities. Similarly, the indicator of the involvement of PWLEs in policy and program development, implementation and evaluations was rated red by most, indicating that the health system still lacked the culture of valuing patients' and PWLEs' inputs. Service user advocates noted this devaluation and decision‐making power imbalance in past studies. The current findings show very little change in this aspect, suggesting a need for targeted interventions to reduce the gap [26].

In domain 2, most indicators were rated amber, indicating increased resource allocations and strategies to deal with resource gaps. Although participants mentioned difficulty rating due to limited, accurate information, indicators like service affordability and differential quality of space/infrastructures were rated red, highlighting areas where structural barriers are apparent.

Most indicators in Domain 3 were also rated as amber. One aspect to note, however, was that most indicators were rated based on participant's experiences and assumptions in the absence of other data. These assumptions and experiences were primarily focused on health workers and practitioners directly interacting with the patients and do not reflect the attitudes of administrators and policymakers who do not directly encounter mental health patients but are key personnel influencing the system practices and cultures. An area of research on structural stigma could be looking into the attitudes of the administrative staff and health system leaders for a clearer picture of the domain. Nevertheless, similar to domain 1, the indicator on the involvement of PWLEs in treatment decision‐making was rated red by most, demonstrating yet again the power imbalances within the healthcare services that can perpetuate structural vulnerabilities [26].

Indicators in Domain 4 had varied ratings; nevertheless, most indicators were rated red. This underscores most participants' concerns that although the government has slowly prioritised mental health as seen in policies and programmes, their implementation and subsequent impact are poor. Even if there is mental health training for primary healthcare workers and budgeting for community mental health programmes, this does not necessarily lead to improved quality and equitable care. This suggests the need for better insight into translating policy documents to reflect improved quality of care and practices [11].

Similarly, ratings from domain 5 indicate that structural stigma and poor quality of care manifest in lower satisfaction with care, lack of ease of access to services, negative interactions with staff, increased financial burden and insufficient information about care. The ratings also highlight the need to routinely collect such information to better understand healthcare systems' functioning and service quality.

### 4.1 Feasibility and Applicability of Using the Structural Stigma Measurement Framework

One way of determining feasibility and applicability is the availability of information or the ability to retrieve the data without undue burden [18]. While indicators in some domains (such as Domain 1 and Domain 2) had readily available information from government reports and publications, most indicators had minimal information that could be identified through the data mapping exercise. The biggest challenges were the unavailability of the information, as it was never recorded, studied or published and gaps in recency, as some included information was from older studies considering very different healthcare systems and did not reflect the current trends [8, 9]. While the KIIs filled the gaps to some extent and provided nuances to the issue of structural stigma, these are reflections of personal experiences, and triangulating the information was complex.

For instance, most amber ratings for indicators (such as excluding mental health from universal health coverage, unavailability of trained human resources and unaffordable services) were due to the government's inclusion of mental health in—BHS, which involves primary healthcare worker training. However, there was no published information on the number of trained health workers, and the key informants were unsure of possible numbers. Nevertheless, the participants mentioned the government's recording system of trained health workers available from health training centres, which, if published annually, could be sourced without undue burden and be easily accessible. Similarly, little to no information was available on funding allocations and mental health service costs, although these were highlighted by most as essential indicators of structural stigma. These information gaps are reflected as a structural barrier in mental healthcare, as they indicate a lack of proactivity in healthcare systems governance to track progress or recognise challenges.

Some indicators, such as ‘less competency of health workers’, ‘involuntary/compulsory treatment of PWLEs’ and ‘negative interactions with health system staff’, received some non‐responses due to limited information from the evidence mapping and the participants' lack of information/experience. This was specifically true when the domains and types of informants did not match. For example, policymakers struggled to rate domains 4 and 5 on the quality of care and PWLE experiences. Meanwhile, PWLEs and health workers found it difficult to rate or provide additional information on domains 1 and 2 regarding the legal framework and resource allocations.

Participants were also asked to reflect on using the RAG rating system as a visual analogue scale to rate each indicator's information and level of structural stigma in the healthcare system. Most participants liked the ratings using colour codes of red, amber and green, as this was universally understood and provided a simple visual representation of performance. For example, policymakers thought this could give easy signs of which indicators were performing poorly, and which were doing better, thus helping them with decision‐making. This was also useful with PWLEs with literacy barriers, as they could easily pick up the colour code‐based ratings. Similar visual analogue scales have been used to measure mental health outcomes among the low‐literacy population in Nepal [27]. Nevertheless, participants felt that ratings based on only three criteria were restrictive, reflected in many undecided ratings. Some participants suggested a 5‐point RAG rating for added nuance while retaining the visual analogue scale for ease.

Essentially, most participants perceived the indicators and the FOCUS‐MHS framework to be acceptable and applicable in Nepal's healthcare systems to measure mental health‐related structural stigma. Participants underlined the framework's usefulness for development agencies and PWLE advocacy networks in identifying structural barriers and discrimination and as an advocacy tool for policymakers/programmers. However, some participants mentioned the need for concise indicators, as some were difficult to rate or collect information on. Indicators such as ‘unavailability of evidence‐based services’, ‘lack of multi‐sectoral collaboration’, and ‘PWLE lack of ease of access to mental health services’ were examples where participants struggled with ratings due to unclear understanding of what the indicators were trying to measure.

Another suggestion regarding the framework's applicability was the need for comparator conditions for some indicators. As the FOCUS‐MHS framework measures mental health‐related structural stigma and discrimination, participants struggled to rate indicators like ‘unaffordable services’ and ‘differential quality of space/infrastructure’ as information mapped onto the indicators exhibiting structural barriers for mental health services. Nepal's health system, in general, is under‐resourced and under‐functioning, with poor coordination between government tiers, delayed fund releases, staff shortages and issues with medicine procurement [28]. In such a low system‐functioning context, participants argued that the poor functioning of mental healthcare services does not reflect structural discrimination. Therefore, they suggested comparing the indicators with other chronic conditions with similar prevalence rates in the community.

### 4.2 Implications and Recommendations for the Application of the Framework in Healthcare Settings

The FOCUS‐MHS framework and its implementation may be relevant in understanding the structural stigma prevalent in the mental healthcare system in other low‐and‐middle‐income countries (LMICs). However, the findings from this study suggest few recommendations and modifications for implementing the FOCUS‐MHS framework step by step to determine the structural stigma against PWLEs in other healthcare systems. A guide and template for adapting and implementing the FOCUS‐MHS can be accessed in File S1.

Determining relevant indicators for different health system settings: Although the indicators within the framework were developed through a comprehensive Delphi exercise with diverse stakeholders representing high, low and middle‐income country settings, not all indicators may be relevant or applicable to all cultural/health system contexts. Nevertheless, the five domains within the framework are meant to provide a comprehensive and nuanced portrayal of mental health‐related structural stigma within the healthcare system and areas that need targeted interventions. These domains also help compare nature and level of structural stigma in different health system settings. Hence, the first step in implementing the framework would be to select the most relevant indicators in each domain.

Data mapping exercise: The data mapping exercise allows researchers to identify information already available from various indicators with information gaps. Information can be collected through multiple sources and methods for better data triangulation.

In Nepal's case, we mapped information from government reports, websites, published directives and peer‐reviewed papers. The mapped information was triangulated with the stakeholder interviews, including collecting any missing information. Other sites could also use existing information sources, such as the WHO Mental Health Atlas [29], WHO Quality Rights reports [22], quality of care, audit reports and patient satisfaction of care reports that already exist in some healthcare settings [30]. Measures such as the ‘stigma cultures in healthcare’, if applied, can provide rich information that can be mapped onto domain 5 (negative experiences of PWLEs) [31]. Identifying data sources and methods of data collection is vital for the quality, validity and trustworthiness of information mapped into the framework.

As recommended by the study participants, a comparison of the indicators with other healthcare conditions would help understand whether the structural issues are disproportionate to mental health conditions or whether they reflect a generally low‐functioning health system. Hence, the data mapping exercise should also compare the state of the indicators for other health conditions. The sites could compare the indicators to other physical health in general or particular health conditions such as diabetes, cardiovascular diseases or other chronic conditions.

Selection of stakeholders: As experienced while applying the framework in Nepal, different stakeholders better understood different domains. For example, domains 1 and 2 were better understood by policymakers, advocates and development agencies than PWLEs and healthcare workers, while domains 3, 4 and 5 were better understood and comfortably ranked by PWLEs, health administrators and health workers. This could be because many indicators were missing information, so the participants were asked to provide it during their KIIs. Participants had no issues providing ratings where rich information was available for the indicators.

**Figure 3:**
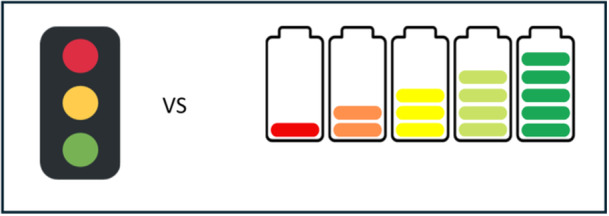
Modification of the visual analogue scale used in rating structural stigma.

Rating exercise: Rating indicators can be done through online surveys or in‐person interviews. The rating may be carried out as a stand‐alone aspect or merged with qualitative interviews, as done in this case example, to understand nuances and complexities within the indicators. This is especially important if the information gap identified from the data mapping exercise is significant. As participants in Nepal suggested, visual analogue rating scales support participants, such as policymakers and PWLEs, in better understanding the criteria and grading how the health systems domains perform regarding structural stigma. One suggested modification is using a 5‐point visual analogue rating scale, like battery level, instead of a 3‐point RAG rating scale (see Figure 3 for the modified visual analogue scale). This gives participants more flexibility to rate the indicators. The criteria for rating based on this visual analogue scale is explained in the recommended guide for the implementation of FOCUS‐MHS (see File S1).

## 5 Conclusion

This study highlights the need for a comprehensive understanding of structural stigma within a mental healthcare system, which we found was applicable and feasible through implementing the FOCUS‐MHS in Nepal's healthcare system, albeit with some modifications.

The stakeholders recognised the potential of FOCUS‐MHS as a valuable tool to track structural stigma in health systems over time and use it as an advocacy tool to guide policy and practice. The study's exploration of the framework's application method, its adaptability to the local context and its emphasis on stakeholder engagement provide a robust foundation for ongoing assessment of structural stigma and discrimination in healthcare settings and aid in developing targeted interventions to reduce structural stigma. The complex measurement methods, findings specific to the Nepal context, subjective ratings and lack of focus on the intersectionality of structural vulnerabilities are some limitations that warrant further exploration.

Nevertheless, the study revealed that while there have been efforts to include mental health in national policies and integrate it within primary healthcare services, substantial barriers remain, including a lack of involvement of PWLEs in decision‐making, inadequate service availability and poor quality of care. Moving forward, health system leaders like policymakers, health workers and development partners need to collaborate with PWLEs and advocates to address these challenges and mitigate the structural stigma seen in the healthcare system in Nepal.

## Author Contributions

**Dristy Gurung:** conceptualization, investigation, writing–original draft, methodology, formal analysis, project administration, writing–review and editing. **Bhawana Subedi:** investigation, writing–review and editing, project administration, formal analysis. **Binita Acharya:** investigation, writing–review and editing, formal analysis. **Mani Neupane:** writing–review and editing, investigation. **Brandon A. Kohrt:** writing–review and editing, visualization, supervision. **Graham Thornicroft:** supervision, writing–review and editing. **Petra C. Gronholm:** writing–review and editing, supervision.

## Ethics Statement

Ethical approval for the study was provided by King's College London Psychiatry, Nursing and Midwifery Research Ethics Subcommittee (reference: HR/DP‐21/22‐24921) and Nepal Health Research Council (reference: 132/2023).

## Conflicts of Interest

The authors declare no conflicts of interest.

## Supporting information

Supporting information.

## Data Availability

Due to confidentiality issues, qualitative transcripts from the participants cannot be openly accessed. Data charted in the Excel sheet can be made available upon request.
