# Supplementary material for: Feasibility and Applicability of Implementing the Framework for Comprehensive Understanding of Structural Stigma in Mental Healthcare Systems: A Case Example of Nepal
Source: Health Expect. 2025 Feb 5;28(1):e70170. doi: 10.1111/hex.70170 (PMC11799572; doi:10.1111/hex.70170)
Supplement: Supplementary file 1 — Supporting information. [file HEX-28-e70170-s001.pdf]

# A guide for assessing mental health-related structural stigma using the FramewOrk for Comprehensive Understanding of Structural Stigma in Mental Healthcare Systems (FOCUS-MHS)

**Who can assess structural stigma?**

This assessment can be carried out by government agencies, development partners, people with lived experience (PWLE) advocacy groups, or by researchers who want to examine structural stigma and discrimination related to mental health in healthcare systems.

**Who can participate in the assessment?**

This assessment is designed to be completed by stakeholders who are part of the healthcare system in various capacities, such as policymakers, PWLE/service users accessing services, advocates, health administrators, healthcare providers, and development/funding partners. Users of this framework can identify the relevant stakeholders based on their site-specific context.

**Does the exercise need to be conducted for all indicators in all the domains?**

Although the indicators within the framework were developed through a comprehensive Delphi exercise with diverse stakeholders representing High / Low and middle-income country settings, not all indicators may be relevant to all settings and their cultural/health system contexts. Nevertheless, the five domains within the framework are meant to provide a comprehensive and nuanced portrayal of mental health-related structural stigma within the healthcare system and areas that need targeted interventions. These domains also help compare the nature and level of structural stigma in different health system settings. Hence, while sites may pick the most relevant indicators in each domain, it would be helpful for sites to try to conduct the exercise for all the domains.

**What are the steps for using this framework?**

Step 1- Determining the relevant indicators in each domain: The site teams could determine and select the indicators from the framework that are most relevant to their healthcare system setting.

Step 2 - Collating information and mapping it to each domain's indicators: Once the indicators for each domain are selected, the next step is to identify information and map it onto the indicators. The information can be identified from various sources, such as published reports/government briefs from government or development agencies such as WHO, or from other relevant studies.

As we are looking at mental health-related structural stigma and discrimination for PWLEs in the healthcare system, comparing the indicators with other healthcare conditions would help us understand whether the structural issues are

disproportionate to mental health conditions or whether the health system is low-functioning in general and does not necessarily reflect structural stigma for PWLEs. Hence, the data mapping exercise should also compare the state of the indicators for other health conditions. The sites could compare the indicators to other physical health in general or choose to compare the indicators to conditions such as diabetes, cardiovascular diseases, or other chronic conditions.

Step 3-Rating exercise: The rating exercise can be conducted through online surveys or in-person interviews with stakeholders identified for the exercise. A visual analogue scale using battery levels may help the participants interpret the rating criteria and visually aid in identifying areas needed for intervention in the healthcare system. The 5-item visual analogue scale and its criteria are described below.

| | |
|---|---|
| 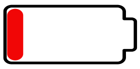 | **RED:** Compared to general healthcare, the mapped information for the indicator represents a **very high level of structural stigma** for mental health within the healthcare system, with no mechanisms in place to reduce it. This may include:<br>• No coverage of mental health in national policies/programs, no allocation of budget and other resources for mental health<br>• No mental health training or training to improve competencies and attitudes among health system personnel<br>• Coercive and fragmented care practices in most institutions without any checks from the health system and have no access to mental healthcare<br>• Overall negative experiences of PWLEs while accessing mental health services from the healthcare system when compared to general healthcare services |
| 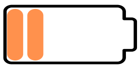 | **ORANGE:** Compared to general healthcare, the mapped information for the indicator represents a **high level of structural stigma** within the healthcare system, with some ad hoc mechanisms in place to reduce it. However, the mechanisms do not necessarily lead to improved structural stigma. This may include:<br>• Some coverage of mental health in national policies/programs but without specific action plans to implement them<br>• Ad hoc allocation of budget and other resources for mental health that is not evidence-based and is not sustainable compared to general healthcare services<br>• Some mental health training or training to improve competencies and attitudes among health system personnel among small groups that have little impact<br>• Culture of coercive and fragmented care practices with some policies/mechanisms in place to improve but not implemented and have low access to mental health care<br>• Minimal improvement in negative experiences of PWLEs while accessing mental health services from the healthcare system when compared to general healthcare services |

| | |
|---|---|
| 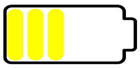 | **YELLOW:** Compared to general healthcare, the mapped information for the indicator represents a **mid-level structural stigma** within the healthcare system, with some evidence-based mechanisms to reduce it. This may include:<br>• Some coverage of mental health in national policies/programs with action plans and strategies to implement them<br>• Planned allocation of budget and other resources for mental health based on need/prevalence and other evidence but is not on par with general healthcare and is not sustainable<br>• Some planned mental health training or training to improve competencies and attitudes among health system personnel<br>• Selected actions and policies that address the culture of coercive and fragmented care practices in the health system are in place and have shown some impacts, leading to better access to care<br>• Some negative experiences of PWLEs while accessing mental health services from the healthcare system, which is not vastly different from accessing other general healthcare services |
| 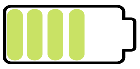 | **LIME:** Compared to general healthcare, the mapped information for the indicator represents a **low-level structural stigma** within the healthcare system, with evidence-based mechanisms to improve it. This may include:<br>• Good coverage of mental health in national policies/programs with practical action plans and strategies that have been implemented widely<br>• Better planning and implementation in the allocation of budget and other resources for mental health based on evidence on need/prevalence that is on par with general healthcare with some mechanisms in place for sustainability<br>• Planned mental health training or training to improve competencies and attitudes among health system personnel that is implemented widely and have shown good impact<br>• Actions and policies are in place to tackle the culture of coercive and fragmented care practices in the health system that have shown good impacts and have led to improved access to care<br>• Experiences of PWLEs while accessing mental health services from the healthcare system are similar to general healthcare services, and while PWLEs may have some negative experiences, it is not explicitly related to their mental health condition |
| 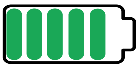 | **GREEN:** The information mapped onto the indicators **does not reflect structural stigma** for mental health conditions when compared to general healthcare within the healthcare system, and evidence-based mechanisms and strategies are in place to address it. This may include:<br>• Mental health is widely covered in national policies/programs with effective action plans and strategies that have been implemented |

| | |
|---|---|
| | widely to overcome any structural barriers to mental healthcare services in the healthcare system<br>• The allocation of budget and other resources for mental health is based on evidence on need/prevalence that is on par with general healthcare with effective mechanisms in place for its sustainability<br>• Mental health training or training to improve competencies and attitudes among health system personnel is integrated widely within the healthcare system's regular programs and has shown excellent outcomes<br>• There is no culture of coercive and fragmented care practices in the healthcare system due to the implementation of actions and policies to tackle them, leading to improved access to care<br>• PWLEs have overall positive experiences while accessing mental health services from the healthcare system, irrespective of their experiences of accessing general healthcare services |

Template for documenting information on mental health-related structural stigma using FOCUS-MHS

| Indicators | Mapping of Relevant information | Information sources | Rating using a visual analogue scale | | | | | Remarks on the indicator and its rating |
|---|---|---|---|---|---|---|---|---|
| | | | 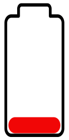 | 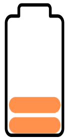 | 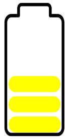 | 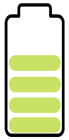 | 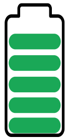 | |
| **Domain 1** | **Poor and discriminatory legal frameworks and policy environment** | | | | | | | |
| Indicator 1 | | | | | | | | |
| Indicator 2 | | | | | | | | |
| Indicator 3 | | | | | | | | |
| Indicator 4 | | | | | | | | |
| Indicator 5 | | | | | | | | |
| **Domain 2** | **Stigmatising system infrastructure and resource allocation** | | | | | | | |
| Indicator 1 | | | | | | | | |
| Indicator 2 | | | | | | | | |
| Indicator 3 | | | | | | | | |
| Indicator 4 | | | | | | | | |
| Indicator 5 | | | | | | | | |
| **Domain 3** | **Aggregate stigma attitude and practices of healthcare system personnel** | | | | | | | |
| Indicator 1 | | | | | | | | |
| Indicator 2 | | | | | | | | |
| Indicator 3 | | | | | | | | |
| Indicator 4 | | | | | | | | |
| Indicator 5 | | | | | | | | |
| **Domain 4** | **Inequitable and poor quality of care** | | | | | | | |
| Indicator 1 | | | | | | | | |
| Indicator 2 | | | | | | | | |
| Indicator 3 | | | | | | | | |

| **Indicators** | **Mapping of Relevant information** | **Information sources** | **Rating using a visual analogue scale** | | | | | **Remarks on the indicator and its rating** |
|---|---|---|---|---|---|---|---|---|
| | | | 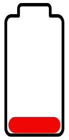 | 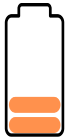 | 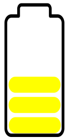 | 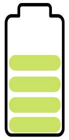 | 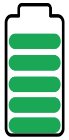 | |
| Indicator 4 | | | | | | | | |
| Indicator 5 | | | | | | | | |
| **Domain 5** | **Negative experiences of PWLEs** | | | | | | | |
| Indicator 1 | | | | | | | | |
| Indicator 2 | | | | | | | | |
| Indicator 3 | | | | | | | | |
| Indicator 4 | | | | | | | | |
| Indicator 5 | | | | | | | | |
